# Transplantation of Umbilical Cord-Derived Mesenchymal Stem Cells Attenuates Surgical Wound-Induced Blood-Brain Barrier Dysfunction in Mice

**DOI:** 10.1155/2023/8667045

**Published:** 2023-02-28

**Authors:** Jie Yang, Hongyu Li, Mingzi Ran, Shuxu Yang, Kui Ma, Cuiping Zhang, Minglu Xiao, Yuguang Yang, Xiaobing Fu, Siming Yang

**Affiliations:** ^1^Research Center for Tissue Repair and Regeneration Affiliated to the Medical Innovation Research Department, PLA General Hospital and PLA Medical College, Beijing, China 100853; ^2^Department of Dermatology, 4th Medical Center, PLA General Hospital, Beijing, China 100048; ^3^Tianjin Medical University, Tianjin, China 300070; ^4^Department of Anesthesiology, 4th Medical Center, PLA General Hospital, Beijing, China 100048

## Abstract

Blood-brain barrier (BBB) is the most important component of central nervous system (CNS) to keep toxins and pathogens from CNS. Although our studies demonstrated that using interleukin-6 antibodies (IL-6-AB) reversed the increased permeability of BBB, IL-6-AB is limited in their application that only could be used a few hours before surgery and seemed delayed the surgical wounds healing process, which urges us to find another more effective method. In this study, we employed the C57BL/6J female mice to investigate the potential effects of umbilical cord-derived mesenchymal stem cells (UC-MSCs) transplantation on BBB dysfunction induced by surgical wound. Compared to IL-6-AB, the transplantation of UC-MSCs more effectively decreased the BBB permeability after surgical wound evaluated by dextran tracer (immunofluorescence imaging and luorescence quantification). In addition, UC-MSCs can largely decrease the ratio of proinflammatory cytokine IL-6 to the anti-inflammatory cytokine IL-10 in both serum and brain tissue after surgical wound. Moreover, UC-MSCs successfully increased the levels of tight junction proteins (TJs) in BBB such as ZO-1, Occludin, and Claudin-5 and extremely decreased the level of matrix metalloproteinase-9 (MMP-9). Interestingly, UC-MSCs treatment also had positive effects on wound healing while protecting the BBB dysfunction induced by surgical wound compared to IL-6-AB treatment. These findings suggest that UC-MSCs transplantation is a highly efficient and promising approach on protecting the integrity of BBB which caused by peripheral traumatic injuries.

## 1. Introduction

Patients who suffered from severe peripheral trauma have been showed cognitive dysfunction, dementia, and other central nervous system (CNS) symptoms around world [1, 2], which often cause impressive economic and social burden [3, 4]. Latest studies indicated that the abnormal function of blood-brain barrier (BBB) plays a critical role for inducing CNS symptoms after injuries [1, 5].

BBB is a delicate neurovascular unit (NVU) relying on the careful reaction of different tissue types including brain microvascular endothelial cells (BMECs), pericyte (PC), astrocyte (AC) end-feet, microglia, and neurons, which prevents pathogens, toxins, and immune cells and their production from infiltrating into CNS to maintain appropriate cerebral functioning [6, 7]. Above all, the disruption of BBB will induce the CNS inflammation and thus deteriorate the progression of the primary or potential neurological diseases. However, most studies focused on BBB impairment induced by cerebral diseases or injuries, but few carried about the influence of peripheral injuries on BBB. Our previous study indicated that BBB permeability could be impaired through peripheral proinflammatory cytokine, interleukin-6 (IL-6) in burns and surgery of mice model [1, 8]. In addition, conditionally using interleukin-6 antibodies (IL-6-AB) could efficiently maintain the normal function of BBB from the inflammatory cytokines infiltrating into CNS [1]. However, the administration of IL-6-AB has some limitations: (a) it takes time for IL-6-AB to neutralize IL-6 in the body, which means it must be used before traumatic surgery to effectively neutralize the corresponding antibodies and thus cannot be used to treat an acute injury. (b) Recent studies show that the administration of IL-6-AB seemed to delay the surgical wounds healing process [9–11]. As a result, the administration of IL-6-AB to maintain the integrity of BBB before traumatic surgery was the only but restrictive method previously. There is an urgency to find an effective and little limited method to treat the traumatic injury.

Mesenchymal stem cells (MSCs) have been showed the therapeutic effects on many diseases and injuries in medical field [12–15]. Specifically, transplantation of MSCs has positive effects both on CNS injuries [16, 17] and peripheral inflammatory diseases [18, 19]. However, there are few studies on the influence of the administration of MSCs on BBB impairment caused by peripheral traumatic surgery at present. Harvesting MSCs can be easily from many resources such as bone marrow (BM), umbilical cord (UC), adipose tissue (ADt), skin, peripheral blood, and placenta. Among these resources, UC-derived MSCs (UC-MSCs), the common attributes of MSCs, have stable and special biological functions including the ability of regulatory inflammation, angiogenesis, relatively easy accessibility, abundant source, and no ethical issues, which make it a perfect choice for this study and future treatment [20]. As a result, in the current studies, we aimed to investigate the overall positive effects of UC-MSCs which have promising ability of modulatory inflammation and regeneration on maintaining the normal function of BBB after traumatic surgical wound.

## 2. Materials and Methods

### 2.1. Mice Surgery and Treatment

Our study performed mice surgical wound model and treatment method which were approved by the PLA General Hospital Standing Committee on the Use of Animals in Research and Teaching. 120 wild-type C57BL/6J female mice about 8-month-old and 1 C57BL/6J pregnant mouse about 20 days (SPF Biotechnology, China) were used in the research.

Four groups were systematically set up including control, surgical wound (S-W), MSC, and IL-6-AB groups: control group without surgery or treatment generally used 21 mice; S-W group with surgery but no treatment used 24 mice; MSC group with surgery and UC-MSCs treatment used 60 mice (1 hrs after surgery 12 mice, 6 hrs before surgery 12 mice, 12 hrs before surgery 24 mice, and 18 hrs before surgery 12 mice); IL-6-AB group with surgery and IL-6-AB treatment used 15 mice. Every experiment was performed twice. The mice were randomly assigned to each group by weight. Mice in S-W, MSC, and IL-6-AB groups were under surgery as described before [1]. The control group was placed in the home cages as same as other groups. The treatments of IL-6-AB and MSCs were exerted as described in our previous studies with a little modification [1, 21]. Briefly, after anesthetizing the mice, a longitudinal incision along the center line was performed to the inside of peritoneum. After performing the trauma, the wound was sutured, and the EMLA cream was smeared to the sutured wound. Each mouse in IL-6-AB group received the 10 *μ*g IL-6-AB at 18 h before the surgery via tail vein injection. Mice in MSCs group received the suspension of UC-MSCs with 1^∗^10^6^ cells in 100 *μ*l phosphate-buffered saline (PBS) at a rate of 0.4 ml/minute through tail vein under brief anesthesia (1% pentobarbital sodium for 5 min) at 6, 12, and 18 hrs before procedure or at 1 hour after procedure. The mice in control group received saline.

### 2.2. Brain Tissue Harvest

Before harvesting the samples, the vessels of all mice were washed with PBS for all studies except the immunofluorescence for BBB permeability. Briefly, after anesthetizing the mice, the heart of each mouse was perfused slowly with PBS until the spilled showed colorless. After lavage, the brain tissues without cerebellar tissue and olfactory bulb will be stored in a −80°C refrigerator for the next studies as previously described with modification [1].

### 2.3. UC-MSCs Harvest and Proliferation

UC-MSCs were harvested as described in previous studies with modification [21, 22]. Briefly, C57BL/6J pregnant murine was sacrificed and then sterilized through 75% alcohol. The abdomen was cut and open, and the embryos (about 8) were separated. Then, umbilical cord tissue per embryo was separated and slightly cut into small pieces. Type I collagenase was performed to digest the harvested tissues in the incubator for 2 hours. Centrifugation was performed to remove the supernatant. Next, we resuspended the cells with Dulbecco's modified eagle medium plus 10% fetal bovine serum (Gibco, USA) and incubated the cells. The medium in the plates was changed every 2 days. The cells grown to almost 80% were passaged. The phenotype of UC-MSCs was assessed by flow cytometry using mouse mesenchymal stem cell phenotype identification kit (Cyagen, USA). The results showed that CD29, CD90, and Sca-1 were positive, and CD31, CD34, and CD117 were negative (Figure S1). The 3^rd^ passage of UC-MSCs was intravenous administered after traumatic surgical wound.

### 2.4. Immunofluorescence

10 kDa dextran was performed to estimate the permeability of BBB after surgical wound as described with modifications [1, 8, 21]. Briefly, at 6 hours after surgical wound, 100 *μ*l PBS with 400 *μ*g 10 kDa dextran tetramethylrhodamine lysine fixable (Invitrogen, USA) was intravenous administered into each mouse, and then, we harvested the brain tissues. The tissues were soaked into 4% paraformaldehyde (PFA) overnight at 4°C and turned into 30% sucrose buffer. After dehydration, the tissues were frozen and cut into sections (12 *μ*m). 0.5% Triton X-100 was performed for permeabilizing and washed with PBS. After permeabilizing, 10% bovine serum albumin (BSA) was added for blocking and then incubated with 1 : 200 isolectin B4 (Invitrogen, USA). The results were observed with the fluorescence microscope.

### 2.5. Fluorescence Quantification

Fluorescence quantification of 10 kDa fluoro-ruby-dextran tracer (Invitrogen, USA) from brain tissues was performed as described in previous research [1, 8, 21]. Briefly, at 6 hours after surgical wound, dextran tracer was intravenous administered into each mouse after traumatic surgical wound. 10 minutes after injection, each mouse was perfused as described. The 10 kDa dextran tracer from the brain tissues after homogenizing was measured by microplate luminescence analyzer.

### 2.6. Enzyme-Linked Immunosorbent Assay

The mouse IL-6/IL-10 immunoassay kit (Andygene, Beijing, China) was performed by the protocol. Briefly, the antibodies were coated onto the wells. The samples were added onto the wells to incubate and washed with wash buffer. At last, the solutions of the kit were added according to the protocol. Analysis of the samples was measured by microplate luminescence analyzer at 450 nm.

### 2.7. Western Blot Analysis

Western blot analysis was performed as described [21]. Tissues were harvested from control, S-W, and MSC groups (12 hours before surgical wound) at 6 hours after surgical wound. Anti-Claudin-5 antibody (Invitrogen, USA), anti-Occludin antibody (Abcam, USA), anti-ZO-1 antibody (Abcam, USA), anti-matrix metalloproteinase-9 (MMP-9) antibody (Abcam, USA), anti-synaptophysin antibody (Abcam, USA), and anti-endothelial nitric oxide (eNOS) antibody (Abcam, USA) were performed in the analysis. The ratio of the levels of targeted proteins to the level of *β*-actin was performed to analyze the trend of those proteins in the brain tissues.

### 2.8. Histological Analysis of Surgical Wound

The different recovery progression after surgical wound was observed among S-W, MSC, and IL-6-AB groups on 3, 7, and 11 days. All mice were sacrificed by neck dislocation method, and the full-thickness slots were harvested. The extracted tissues were soaked with 4% PFA at 4°C overnight. After fixing, the samples were embedded into paraffin and cut into 6 mm sections. Hematoxylin and eosin (H&E) was performed to assay the progression of wound healing. Measurement of wound depths was performed as previously described with modification [23]. Briefly, the Image J software was performed to measure the neoepidermal thickness (from the basement membrane to the surface of the epidermis) and the reepithelization (reepithelization = [(distance on day 0–distance on indicated day)/(distance on day 0)] × 100%).

### 2.9. Statistical Analysis

One-way and two-way ANOVA were performed in each study to analyze the difference of group. *p* values less than 0.05 were considered statistically significant. All statistical data was analyzed by Prism 8 software.

## 3. Results

### 3.1. Transplantation of UC-MSCs Alleviate Extravascular Dextran Level Induced by Surgical Wound

The phenotype of UC-MSCs was assessed by flow cytometry. The results showed that CD29, CD90, and Sca-1 were positive, and CD31, CD34, and CD117 were negative (Figure S1), which was consistent with the phenotype of mouse MSCs. In addition, the microscopic features of sample cells were turned into fusiform, and adhesion of those was increased from primary passage to the 3rd passage, which was consistent with the morphological characteristics of mouse MSCs (Figure S2). Immunofluorescence with blood vessels (green color) and 10 kDa dextran (red color) was performed to evaluate whether intravenous administration of UC-MSCs could alleviate the permeability of BBB induced by surgical wound and the optimal time point for treatment. As compared to control group, the level of 10 kDa tracer level outside the BMECs in the brain tissues (cortex) obviously increased in S-W group, while the UC-MSCs could effectively alleviate the leakage of BBB at 6, 12, and 18 hours before or at 1 hour after the same procession compared it to control group. The results indicated that the permeability of BBB was increased by surgical wound and UC-MSCs can effectively alleviate extravascular dextran level before or after surgical wound.

**Figure 1:**
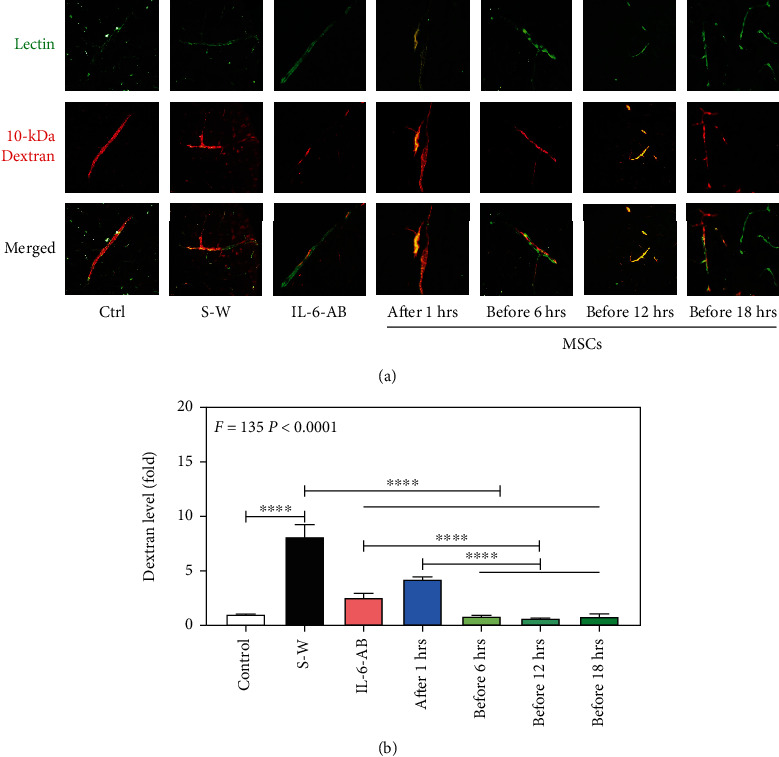
UC-MSCs alleviated extravascular dextran level at different time points induced by surgical wound in mice. (a) The blood vessels were showed green at the first line, and dextran was showed red at the second line. The brain section of control group (the first column), the S-W (surgical wound group) after 6 hours (the second column), IL-6-AB after surgical wound (the third column), UC-MSCs at 1 hour after surgical wound (the fourth column), UC-MSCs at 6 hours (the fifth column), 12 hours (the sixth column), and 18 hours before surgical wound (the seventh column). The red spots of nonoverlap showed in the third line indicate the dextran outside the BMECs. *N* = 6 in each group. Scale bar, 50um. (b) Fluorescent quantitation showed that different time points of UC-MSCs and IL-6-AB decreased extravascular level of 10 kDa dextran outside the BMECs compared with the S-W group (black bar) after surgical wound, and the most efficient level is the administration of UC-MSCs before surgical wound. *N* = 6 in each group (^∗^for comparing with control, ^∗∗^*p* < 0.01, ^∗∗∗^*p* < 0.001, ^∗∗∗∗^*p* < 0.0001).

Secondly, we performed fluorescent quantification to further evaluate the capability of UC-MSCs in protecting BBB integrity in vivo. We found that compared to S-W group, transplantation of UC-MSCs both before and after surgical wound could effectively protect BBB from disruption. Moreover, the extent of the level of 10-kDa dextran outside the BMECs in pretreated UC-MSCs at 6, 12, and 18 hours had the best effects on protecting the integrity of BBB compared to S-W group (Figure 1(b)) and was almost the same as the level of that in control group (Figure 1(b)). Finally, the therapeutic efficiency of preinjection of UC-MSCs was better than that of postsurgery injection and traditional (IL-6-AB) groups (Figure 1(b)). These results further indicated that the permeability of BBB was increased by surgical wound, and transplantation of UC-MSCs in vivo had no time limitation to protect BBB dysfunction induced by surgical wound, while the best time point to pretreat with UC-MSCs was at least 6 hours before surgical wound.

### 3.2. UC-MSCs Decrease the IL-6 and Increase the IL-10 Levels in Blood and Brain Tissue

**Figure 2:**
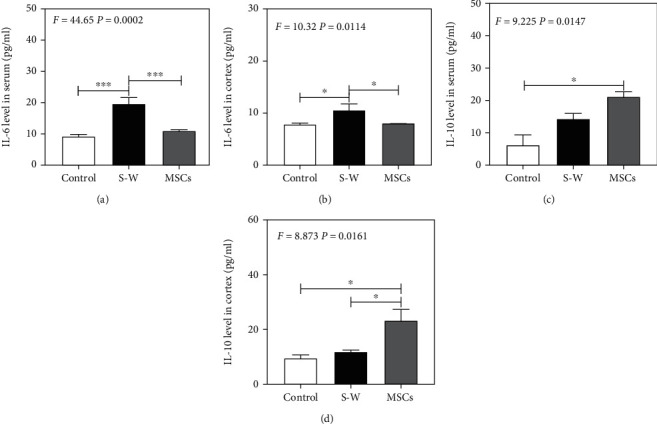
The expression of IL-6 and IL-10 in serum and cortex at 6 hours after surgical wound and the influence of UC-MSCs. (a, b) The expression of IL-6 at different groups after surgical wound in serum and cortex. (c, d) The expression of IL-10 at different groups after surgical wound in serum and cortex. *N* = 3 in each group (^∗^*p* < 0.05, ^∗∗∗^*p* < 0.001).

Given the previous findings that both blood and brain IL-6 level was increased by surgical wound in mice [1], next, we asked the influence of UC-MSCs on the proinflammatory cytokine IL-6 and the anti-inflammatory cytokine IL-10 levels. We found that compared with the control group (Figures 2(a) and 2(b)), surgical wound increased IL-6 level in blood and cortex at 6 hours (Figures 2(a) and 2(b)) which proves our preceding findings, while UC-MSCs (12 hours before surgery) decreased IL-6 level in blood and brain at 6 hours after surgical wound compared with the control group (Figures 2(a) and 2(b)); UC-MSCs (12 hours before surgery) increased IL-10 level in blood and brain at 6 hours after surgical wound (Figures 2(c) and 2(d)). These results indicated that transplantation of UC-MSCs can effectively maintain the integrity of BBB by attenuating the ratio of IL-6 to IL-10 in serum and cortex and thus attenuate neuroinflammation induced by surgical wound.

### 3.3. UC-MSCs Increase the Expression of TJs and Decrease the Expression of MMP-9 after Surgical Wound

**Figure 3:**
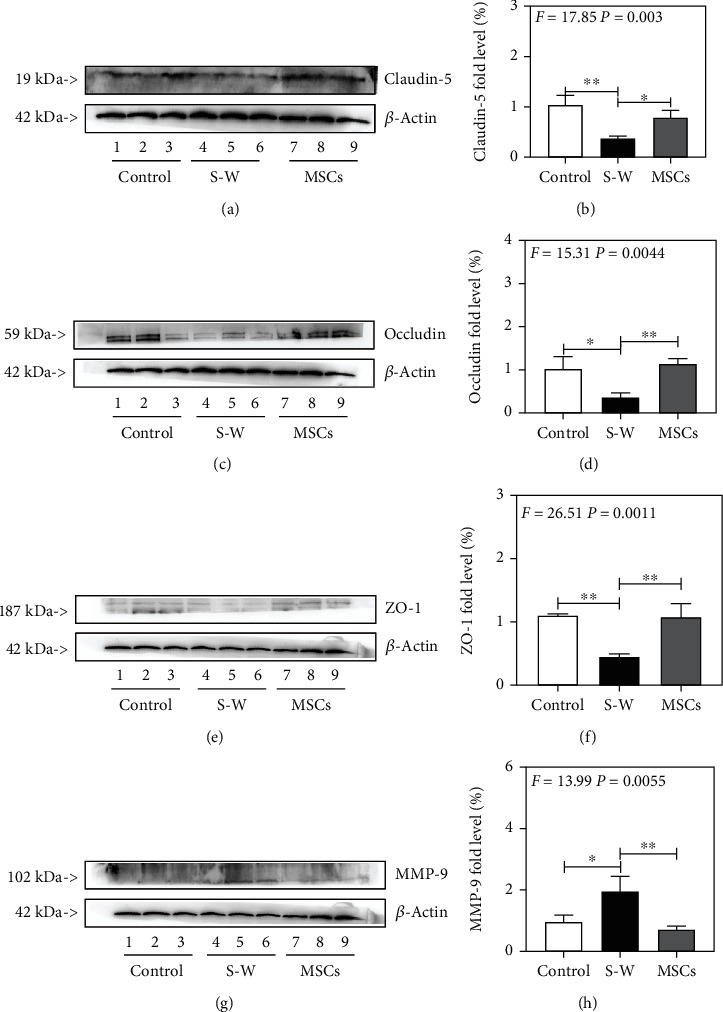
The expression of TJs and MMP-9 in BBB after surgical wound in control, S-W, and MSCs (12 hours before surgical wound) groups. (a, b) The expression of Claudin-5 at 6 hours after surgical wound in control, S-W, and MSCs (12 hours before surgical wound) groups. (c, d) The expression of Occludin at 6 hours after surgical wound in control, S-W, and MSCs (12 hours before surgical wound) groups. (e, f) The expression of ZO-1 at 6 hours after surgical wound in control, S-W, and MSCs (12 hours before surgical wound) groups. (g, h) The expression of MMP-9 at 6 hours after surgical wound in control, S-W, and MSCs (12 hours before surgical wound) groups. *N* = 3 in each group (^∗^*p* < 0.05, ^∗∗^*p* < 0.01).

Given the findings that surgical wound could disrupt the integrity of BBB and the transplantation of UC-MSCs could attenuate it, next, we compared the therapeutic effects of UC-MSCs on the TJs, including Claudin-5, Occludin, and ZO-1. Western blot assay showed that the expression of TJs was extremely decreasing after surgical wound including Claudin-5 (Figures 3(a) and 3(b)), Occludin (Figures 3(c) and 3(d)), and ZO-1 (Figures 3(e) and 3(f)), while UC-MSCs (12 hours before surgery) obviously improved the decreasing expression of TJs compared with S-W group at 6 hours in mice. Furthermore, we detected the level of MMP-9, a member of main protease family with the ability to increasing the permeability of BBB and was positive correlation with the expression of IL-6 [24–26]. Western blot assay indicated that compared with the control group, surgical wound increased the expression of MMP-9, while transplantation of UC-MSCs decreased that after surgical wound (Figures 3(g) and 3(h)). These results indicated that the administration of UC-MSCs could alleviate the disruption of BBB by preventing the decreasing expression of TJs and the basal membrane from digesting by downregulating the expression of MMP-9.

### 3.4. UC-MSCs Promoted the Processes of Surgical Wound Healing

**Figure 4:**
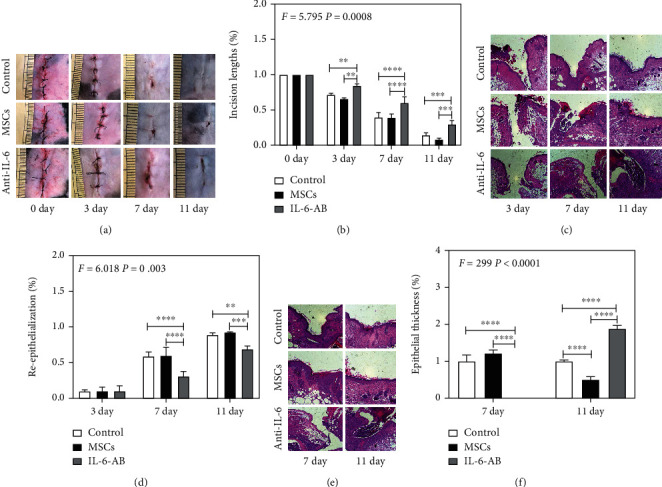
UC-MSCs accelerated surgical wound healing in mice. (a) Representative photographs for the progression of surgical wound healing from days 0 to 11. (b) The statistical results of incision length. (c) H&E staining images of tissues at days 3, 7, and 11 after surgical wound. (d) The statistical results of the epidermal depth. (e) H&E staining images of tissues at days 7 and 11 after surgical wound. (f) The statistical results of the thickness of the neoepidermis. *N* = 3 in each group; scale bar, 100 *μ*m (^∗∗^*p* < 0.01, ^∗∗∗^*p* < 0.001, ^∗∗∗∗^*p* < 0.0001).

Three groups, including MSCs (pretreated at 12 hours before surgical wound), IL-6-AB, and control groups (surgery without any treatment), were performed to detect whether UC-MSCs have side effects on wound healing. Observation of abdominal incisions showed that surgical wound in the MSCs and control groups were healing better and less infection than that of the IL-6-AB group (Figure 4(a)). The lengths of incision in both MSCs and control groups were healing better after 3, 7, and 11 days than that of the IL-6-AB group (Figure 4(b)). H&E was performed to detect the reepithelialization of each group. Consistent with the results of surgical wound observation, despite little obvious differences of reepithelialization 3 days after surgical wound, the reepithelialization of recovery in MSCs group was as same as the control group while IL-6-AB group did worse than those did on 7 and 11 days (Figures 4(c) and 4(d)). The depths of wound in MSCs and control groups were significantly better in 7 and 11 days than that of the IL-6-AB group (Figures 4(c) and 4(d)). Moreover, the reepithelialization occurred earlier in both MSCs and control groups than in the IL-6-AB group (Figures 4(e) and 4(f)). On the 7^th^ day after surgical wound, the neoepidermis in the MSCs and control groups were apparent while the IL-6-AB group did not show the way totally. Furthermore, 11 days after surgical wound, the thickness of the neoepidermis in the MSC group were thinner than control and IL-6-AB groups while the thickness of IL-6-AB group was thicker than other groups (Figures 4(e) and 4(f)). This reason may be attributed to the complete maturation of epidermis in MSC group and the incomplete progression in control and IL-6-AB groups. These data showed that transplantation of UC-MSCs promoted the progression of wound healing process while protecting BBB integrity after surgical wound.

## 4. Discussion

Recent studies indicate that the integrity of BBB has been shown to play a critical role on the outset and progression of neurological diseases. The disruption of BBB often predicts the neuroinflammation which would induce or deteriorate the progression of neurological diseases [27, 28]. However, most studies primarily devoted to research the BBB dysfunction induced by CNS injuries or diseases but few noticed the destructive effects induced by peripheral injuries. Our previous studies used burns, and surgical wound in mice indicated that inflammatory cytokines in serum including IL-6 and IL-1*β* can increase the permeability of BBB and thus induced the neuroinflammation [1, 21]. Simultaneously, the administration of IL-6-AB before traumatic surgical wound can effectively maintain the integrity of BBB after the surgery. However, the administration of IL-6-AB has some limitations: (a) the administration of IL-6-AB is effective only when administered a few hours before trauma, which means that IL-6-AB cannot be administered to treat acute injury. (b) Recent studies indicate that using IL-6-AB seemed to delay the surgical wounds healing process [9–11]. As a result, a brand new and effective width method should be explored to meet the requirements in wound healing field.

As demonstrating the therapeutic effects of MSCs to protect the integrity of BBB after traumatic surgical wound in a mouse model, the limitations of mice model need to be elucidate that there are indeed some functional differences between the immune systems of laboratory mice and humans. For example, laboratory mice are more resistant to certain toxins than humans and the acceleration of wound healing. It is true that the results of animal experiments have certain limitations, but we first verify the mechanism in the mouse model to ensure that the theoretical mechanism basis is reliable and repeatable. The next step is gradually to move to animals that are closer to humans and finally to clinical implementation.

In the present study, we used dextran tracer to detect the leakage of BBB and the influence of UC-MSCs transplantation on protecting the integrity of BBB after traumatic surgical wound. We found that surgical wound could increase the permeability of BBB, and the administration of UC-MSCs both before and after surgical wound could effectively protect the integrity of BBB. Although the therapeutic effects of UC-MSCs after surgical wound was a little lower than that of IL-6-AB group, the administration of UC-MSCs before surgical wound groups was much higher than that of both S-W after surgical wound and IL-6-AB groups. Given the fact that the intravenous administration of UC-MSCs could effectively maintain the integrity of BBB within 1 hour after traumatic surgery, these interesting results indicated that UC-MSCs could be used to protect BBB after unpredictable acute cases such as burns and severe accidental cuts. However, preoperative interventions are still recommended in selective surgery or predictable injuries based on these results.

Given the preceding findings that UC-MSCs could effectively improve the disruption of BBB, next, we further determined the potential mechanism. Increasing expression of IL-6 in serum, as our previous study confirmed, played an important role on disrupting the integrity of BBB [1], and some studies also suggest that the proinflammatory role of IL-6 is functioned through activating STAT3 pathway [29, 30]. Interestingly, as same as activating the STAT3 pathway, IL-10 works as an anti-inflammatory role to inhibit the extending inflammation [31], which may indicate that IL-10 can inhibit the inflammation through inhibiting IL-6. As a result, in our current study, we detect the therapeutic effects of UC-MSCs on both inflammatory cytokines, IL-6 and IL-10, in serum and cortex after surgical wound. The data showed that UC-MSCs could decrease IL-6 and increase IL-10 levels in both serum and cortex, which may predict that the administration of UC-MSCs could maintain the integrity of BBB by regulating the ratio of the levels of IL-6 to IL-10. However, in our current study, we suggest that the neutralization of peripheral IL-6 by UC-MSCs could completely protect the integrity of BBB by maintaining the levels of TJs between BMECs and thus avoid the neuroinflammation. Interestingly, the reason why the transplantation of UC-MSCs increased the level of IL-10 in cortex without inflammatory condition remains unknown and needs further studies to elucidate.

Furthermore, we performed Western blot assay to detect the influence of UC-MSCs on TJs in BBB. Western blot assay showed that surgical wound decreased the levels of TJs, while UC-MSCs inhibited the decreasing levels of TJs. These results showed that UC-MSCs could effectively improve the disfunction of BBB by attenuating the decreasing levels of TJs after surgical wound. In addition, MMP-9 is the major contributor to BBB disruption since it can digest the TJs and basal lamina of BMECs, which plays an important role in the increased permeability of BBB under inflammatory condition [25, 26]. In our present study, the results showed that the expression of MMP-9 was extremely increased and UC-MSCs effectively decreased the expression of MMP-9 after surgical wound. These results indicated that UC-MSCs can effectively maintain the TJs and basal lamina of BMECs from digesting by MMP-9 and thus prevent the dysfunction of BBB after surgical wound.

Finally, side effects on primary injury should be considered for a mature treatment. Specific to this study, the quality of surgical wound healing should also be paid attention to. Our previous research was committed to demonstrating the disruption of BBB by peripheral inflammatory factors after traumatic surgical wound and the administration of IL-6-AB through mice tail vein could improve the disruption of BBB in mice abdominal surgery model [1]. However, IL-6-AB seemed delayed the wound healing process, and real extent has not been estimated [9, 10]. Some reports show that MSCs can improve the wound healing [32–35]. As a result, in current research, we also aimed to evaluate the therapeutic effects of UC-MSCs on wound healing after traumatic surgical wound. Although the appearance of UC-MSC group showed little differences from control group, the data on 11 days of epithelial thickness suggests that the transplantation of UC-MSCs has a slight promotion on the process of surgical wound healing compared with control group while protecting the integrity of BBB. However, to achieve the best therapeutic effects of UC-MSCs transplantation on the process of wound healing during preventing the dysfunction of BBB, our future studies need to performed different injecting time points, various frequency, and dosage of UC-MSC transplantation to find out the best method provided for future clinical treatment. In addition, by comparing the effects of UC-MSCs and IL-6-AB on protecting BBB from disruption and promoting the wound healing, respectively, UC-MSCs have performed better on therapeutic effects than IL-6-AB has done, which may provide a promising treatment for future studies.

## 5. Conclusion

The transplantation of UC-MSCs could maintain the TJs to maintain the integrity of BBB and thus avoid the occurrence of neuroinflammation by attenuating the ratio of IL-6 to IL-10 and the expression of MMP-9. More interestingly, the method we performed in the current study tends to promote the process of surgical wound healing while protecting BBB dysfunction induced by surgical wound. These effects made by UC-MSCs may suggest that UC-MSCs is a novel, flexible, and highly efficient approach on protecting BBB dysfunction induced by surgical wound and provide a promising approach for future studies.

## Data Availability

All data generated or analyzed during this study are included in this published article.

## Supplementary Materials

Supplementary Materials S-Figure 1: the flow cytometry analysis of UC-MSCs. The positive mesenchymal markers include CD29, CD90, and Sca-1, and the negative markers include CD31, CD34, and CD117, which were consistent with the phenotype characteristics of mouse MSCs. S-Figure 2: the shapes of UC-MSCs from primary passage to the 3rd passage, respectively. The microscopic features of sample cells were turned into fusiform, and adhesion of those was increased from primary passage to the 3rd passage, which was consistent with the morphological characteristics of mouse MSCs.
