# Supplementary material for: Transplantation of Umbilical Cord-Derived Mesenchymal Stem Cells Attenuates Surgical Wound-Induced Blood-Brain Barrier Dysfunction in Mice
Source: Stem Cells Int. 2023 Feb 28;2023:8667045. doi: 10.1155/2023/8667045 (PMC9991482; doi:10.1155/2023/8667045)
Supplement: Supplementary Materials — S-Figure 1: the flow cytometry analysis of UC-MSCs. The positive mesenchymal markers include CD29, CD90, and Sca-1, and the negative markers include CD31, CD34, and CD117, which were consistent with the phenotype characteristics of mouse MSCs. S-Figure 2: the shapes of UC-MSCs from primary passage to the 3rd passage, respectively. The microscopic features of sample cells were turned into fusiform, and adhesion of those was increased from primary passage to the 3rd passage, which was consistent with the morphological characteristics of mouse MSCs. [file 8667045.f1.docx]

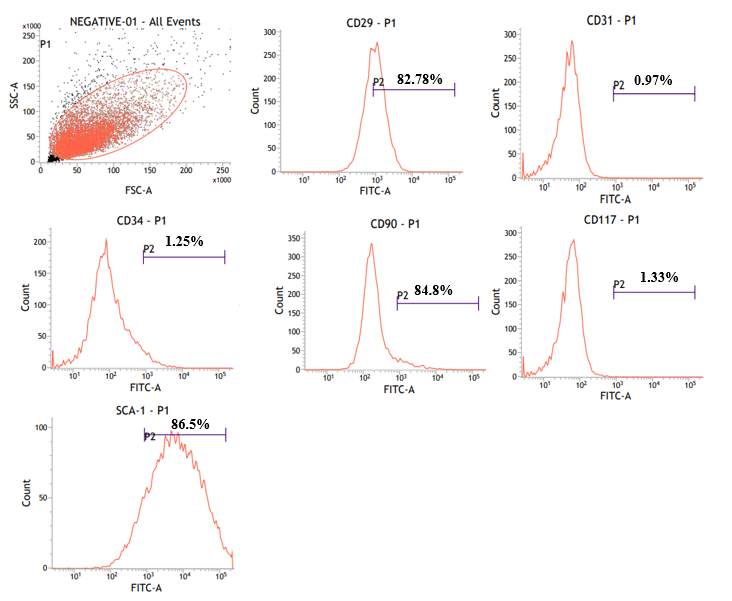


**S-Figure 1. The flow cytometry analysis of UC-MSCs.** The positive mesenchymal markers include CD29, CD90 and Sca-1, the negative markers include CD31, CD34 and CD117.


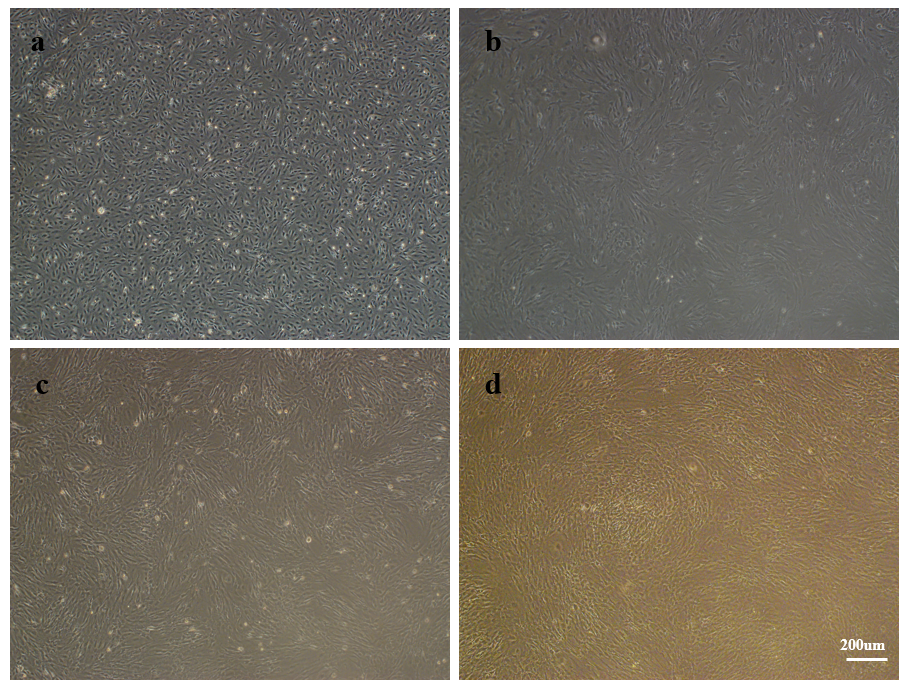


**S-Figure 2. The shape of UC-MSCs. (a).** The shape of primary mesenchymal stem cells. **(b).** The shape of passage one mesenchymal stem cells. **(c).** The shape of passage two mesenchymal stem cells. **(d).** The shape of passage three mesenchymal stem cells.
